# Spectrophotometric analysis of lipid used to examine the phenology of the tick *Ixodes ricinus*

**DOI:** 10.1186/s13071-018-3102-3

**Published:** 2018-09-20

**Authors:** Swaid Abdullah, Saran Davies, Richard Wall

**Affiliations:** Veterinary Parasitology & Ecology Group, School of Biological Sciences, University of Bristol, 24 Tyndall Avenue, Bristol, BS8 1TQ United Kingdom

**Keywords:** *Ixodes ricinus*, Abundance, Phenology, Blood meal, Diapause, Feeding

## Abstract

**Background:**

Ticks store lipid as an energy souce, which depletes progressively between blood meals. The amount of lipid and rate of lipid depletion can be used as a good indicator of the feeding history and assist in explaining the phenology of tick populations. However, existing gravimetric approaches to lipid measurement are relatively imprecise. To improve our ability to accurately measure lipid accumulation and metabolism in individual ticks, a microquantity colorimetric sulfophosphovanillan method of lipid estimation was standardised and used to explore the seasonal variations in the lipid content of *I. ricinus* nymphs.

**Results:**

Lipid values for field-derived questing ticks, collected by blanket dragging, varied between 5–45 μg and clear patterns of lipid depletion were demonstrated under controlled laboratory conditions. For field populations collected monthly over two years, the results indicate that two different cohorts of nymphs enter the questing tick population in autumn and spring, with very few nymphs joining the population in summer.

**Conclusions:**

The data illustrate the seasonal change in lipid content of nymphal ticks, reflecting their feeding history and highlight the utility of the spectrophotometric technique for analysis of lipid in ticks in helping to improve our understanding of seasonal activity patterns.

## Background

In higher latitudes, *Ixodes* ticks typically complete their life-cycle in three years, but the rate of development and patterns of seasonal activity are highly variable and depend upon a complex interplay between photoperiod, temperature, moisture and the availability of hosts [1–3]. When conditions of temperature and humidity are suitable, they quest for as long as their energy reserves last until a host is found, although during this period they may have to retreat to the moist layer of vegetation to prevent desiccation. In areas where humidity is high, such as dense woodland, seasonal tick activity may last for several months, whereas in drier more open areas, water loss and energy depletion are greater, so the period of activity is shorter and may last for only a few weeks [4, 5]. Hence, the onset, duration and timing of seasonal activity varies from region to region depending on the habitat and climate of the region and also the weather during any particular year. The pattern of activity is also affected by life-cycle stage, since they have different thresholds for temperature-dependent activity and dessication; larvae remain close to the moist ground, where they feed on small rodents and can survive and quest for longer in summer than other life-cycle stages [2].

To attempt to explain the complex phenology of tick populations in the field, a critical problem is determining when they last fed and therefore assigning them to any specific feeding cohort. Previous studies have used histological or anatomical examination of ixodid ticks to categorise them into feeding cohorts [6, 7] or have used an understanding of the climatic requirements of different life-cycle stages to infer their previous feeding history from seasonal patterns of abundance. These approaches provide relatively imprecise or indirect conclusions and interpretation may be particularly difficult for regions with highly variable seasonal weather patterns [8]. One approach to providing a more direct, quantitative analysis of the feeding history of an individual tick is through the analysis of its lipid reserves. Ticks store lipid, which is derived from the blood on which they feed. A tick utilises this lipid reserve progressively to support its survival between blood meals, questing activity and reproduction [9, 10]. The measurement of lipid therefore allows the feeding history of an individual tick to be identfied. Lipid reserves are thought to decline relatively predictably with time since the last meal [3]. The lipid reserves may also allow the causes of variation in the seasonal activity pattern seen within tick populations [11] to be understood in greater detail [12]. However, to date, the techniques used for the measurement of lipid levels in ticks have been relatively inexact.

Some previous studies have categorised ixodid ticks simply into residual blood meal or lipid-related age-classes [6, 7]. In contrast, other authors have used a gravimetric method for lipid estimation in the tick, *Ixodes ricinus*. For this, lipid was extracted from entire ticks by weighing them, dipping in 3 consecutive washings of chloroform for 72 h and reweighing [3, 12]. Using this approach, lipid values were used to explain the seasonal dynamics of *I. ricinus* through different seasons of the year [3]. It was reported that a cohort of newly-moulted nymphs usually appeared in autumn and these nymphs were high in lipid content; nymphs with relatively low lipid contents were present during the spring season; these were assumed to be nymphs that had failed to feed the previous autumn. After moulting to become nymphs, the lipid content of ticks declined steadily and if a tick failed to feed, its lipid levels reached a level where it was assumed to have died [3]. However, the description of the gravimetric lipid measurement method used in these studies lacks critical detail and no evaluation of its accuracy was presented. Such an approach is also difficult to use accurately with individual ticks, limiting its utility*.*

Further technological refinement is required to develop a robust, easy-to-use approach to lipid analysis that is accurate enough to use with individual ticks, allowing it to be more widely used by researchers. The aim of the work described here, therefore, was to evaluate a sensitive and accurate spectrophotometric method of lipid estimation for use with individual ticks, adapting a technique first proposed by Van Handel [13] and to demonstrate the use of this approach in interpreting seasonal tick phenology.

## Methods

### Tick collection

*Ixodes ricinus* nymphs were collected from a municipal public park in south-west England, UK [14]. This park is composed of 344 hectares of grassland and woodland located close to the west of the city of Bristol, UK (51°44'79"N, 2°64'46"W) at an altitude ranging between 10 and 120 m above sea level. It presents a variety of habitats, including woodland, semi-improved and unmanaged grassland, public amenity areas, ponds and two deer parks. The park has a resident population of managed red and fallow deer and unmanaged populations of wild roe deer. Only nymphs were collected in this study.

Ticks were collected by the standard procedure of blanket dragging [15]. A 1 m^2^ sheet of white cloth, attached to a wooden stick with a 3 m cord tied to either ends of the stick was used for tick collection. The cloth was dragged slowly over the vegetation so that the cloth remained in contact with the vegetation throughout the dragging process. After each approximately 10 m drag, the cloth was turned, and all the ticks attached to the cloth were collected with fine forceps and stored in a collection vial. The vial was labelled with the date of collection and the ticks were immediately taken to the laboratory and stored at -20 °C to kill them and to preserve them for further processing.

### Lipid extraction, measurement and callibration

A pooled sample of 108 nymphal ticks, collected over several months between 2015 and 2016, was first used to evaluate and refine the lipid extraction technique. For this, after removal from the freezer, ticks were dried at 70 °C for 12 h and then weighed using an ultrasensitive microbalance (Sartorius-ME5, Goettingen, Germany) to the nearest microgram. Lipid was estimated using a spectrophotometric method based on the microquantity colorimetric sulfophosphovanillan method (SPV) for total lipids first described by Van Handel [13]. The technique is based on the principle that unsaturated lipids react with sulphuric acid to produce a carbonium ion, vanillin reacts with phosphoric acid to produce an aromatic phosphate ester, and the carbonium ion reacts with the activated carbonyl group of phospho-vanillin to produce a charged, coloured complex that is stabilized by resonance and absorbs light maximally at about 525 nm [16].

Each individually-dried and weighed tick was placed into a separate clean glass test-tube and crushed with a glass rod in 0.5 ml of a 1:1 chloroform-methanol mixture. The tube was then gently shaken to include any tick debris that had accumulated on its side, then half of the volume (0.25 ml) from this tube was carefully transferred to a second clean glass tube. This tube was then placed in heating block at 100 °C in a fume cupboard to evaporate off the solvent. After the solvent had evaporated, 0.1 ml of sulphuric acid was added, and the tube was heated again for 10 min at the same temperature. The tube was left to cool and after cooling, vanillin reagent was added to make a total volume of 2.5 ml. The vanillin reagent used here was prepared by dissolving 600 mg vanillin in 100 ml hot water and 400 ml of 85% phosphoric acid and stored in the dark. After adding vanillin to the tube, a reddish colour developed in 5 min then slowly faded after 30 min. The tube was read 5 to 10 min after the vanillin had been added in a spectrophotometer (Biochrome, Biowave II, Cambridge, UK) at 525 nm against a blank reagent (neat vanillin reagent) and the lipid content was read directly from a calibration curve.

A calibration curve was obtained by using seven dilutions: 0, 12.5, 25, 50, 100, 200 and 400 μl, of analytical standard soybean oil solution (0.917 g/ml) (Sigma-Aldrich, Gillingham, UK) diluted in methanol: chloroform (1:1) and subjecting them to the procedure as described above. Absorbance higher than 1.00 was reread at 490 nm and the reading was then doubled to get the absorbance values for 525 nm, following the procedure of Van Handel [13].

To allow the lipid reserves recorded to be related to an approximate time between blood meals, a batch of approximately 300 nymphs was collected in May 2017. This batch was collected relatively late in the UK tick season to ensure that a heterogeneous range of feeding histories would be obtained. These ticks were placed in 25 × 25 × 10 cm plastic boxes containing a 1 cm deep base of damp sand and maintained in a cooled incubator at 15 °C and 80% relative humidity (RH). This environment was adequate to ensure good levels of survival of the ticks during the trial. Batches of live ticks were then removed from the boxes for lipid analysis 9, 39, 92 and 239 days after collection to quantify changes in lipid associated with exhaustion of reserves and starvation.

The spectrophotometric method of lipid analysis was compared to a gravimetric method. For this, eight nymphs of *I. ricinus* were dried overnight at 70 °C, weighed and then washed in three 24 h changes of chloroform to remove the lipid, re-dried in the oven at 70 °C again for 24 h and reweighed to get the lipid free body weight, as described by Randolph et al. [3]. The chloroform-treated nymphs were subsequently subjected to the spectrophometric method, as described above, to determine whether any residual lipid remained.

### Seasonal pattern of lipid use

For analysis of the use of the spectrophotometric lipid measurement to examine seasonal patterns of lipid reserves, questing *I. ricinus* nymphs were collected monthly for two years by blanket dragging, as described above. Tick collections started in January 2015 and continued until December 2016 (except for August 2016, when no samples were collected). All the ticks were collected from the same area in the public park described above. On each sampling occasion between 25 and 60 nymphs were collected to provide a statistically meaningful sample for lipid analysis. The collections made each month were analysed separately.

### Statistical analysis

For analysis of changes in lipid values over time, the data were square root transformed to normalise the distribution and subjected to analysis of variance (ANOVA) and Tukey’s multiple comparisons *post-hoc* tests (Statgraphics, 16.1, StatPoint technologies Inc.). Analysis of variance was also used to examine differences in lipid values between homogeneous groups of ticks within samples, identified by nearest neighbour cluster analysis. Lipid contents are plotted as untransformed values for clarity.

## Results

### Lipid extraction, measurement and callibration

**Fig. 1:**
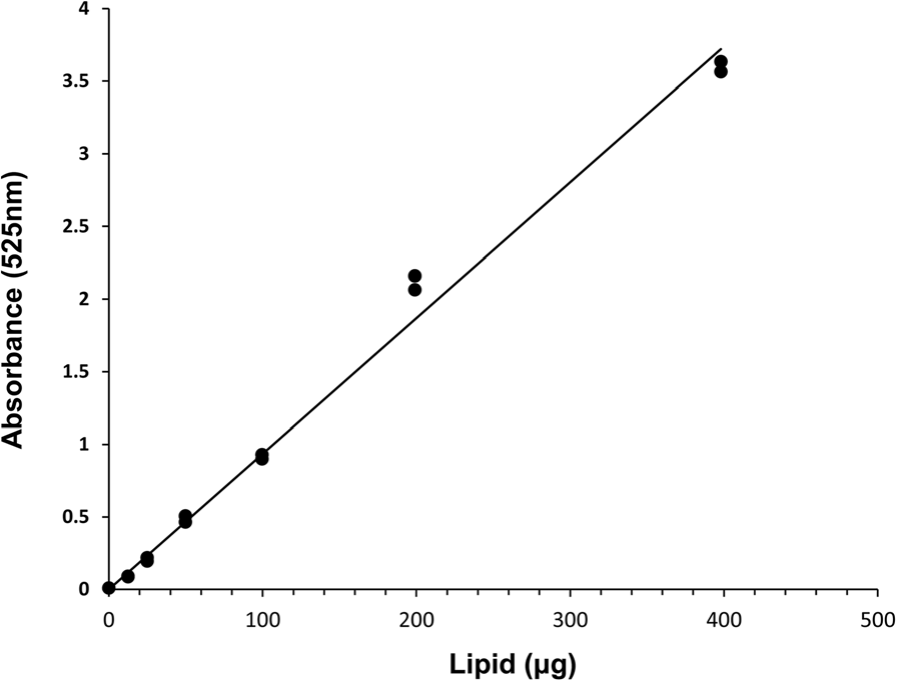
Spectrophotometric absorbance of known concentrations of soybean oil at 525 nm to give a calibration curve. Linear regression fitted: y = 0.0093x, *P* < 0.001, *R*^2^ = 0.991

**Fig. 2:**
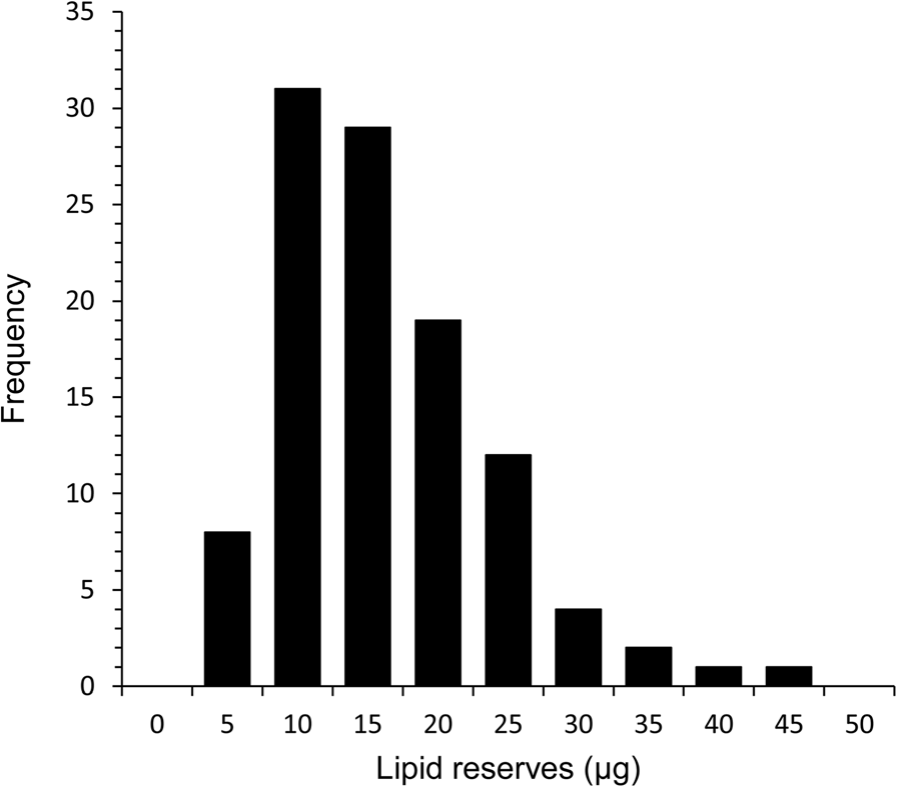
The frequency distribution of observed lipid values (μg), measured by spectrophotometric analysis, in a sample of 100 nymphal *Ixodes ricinus* collected between between 2015 and 2016 in 5 μg lipid classes

The analysis of the soybean standard gave a strong linear relationship between absorbance and lipid concentration showing accuracy and precision to the lowest concentrations examined (Fig. 1). Comparison of the gravimetric and spectrophotometric extraction and measurement methods showed that even after 72 h treatment with chloroform, a mean ± SD of 0.98 ± 0.32 μg of lipid remained in each tick. Hence, the gravimetric technique underestimated lipid content by a mean of 17.17 ± 7.79%. For the pooled sample of 108 nymphs used initially to explore the utility of the technique, the median lipid value was 12.2 μg with a range between a minimum of 1.77 μg and a maximum of 40.8 μg; the frequency distribution was significantly positively skewed (skewness = 4.86, *df* = 106, *P* = 0.001), with 68% of the ticks analysed having lipid reserves of below 15 μg (Fig. 2) and 8% with lipid reserves of 30 μg and above.

**Fig. 3:**
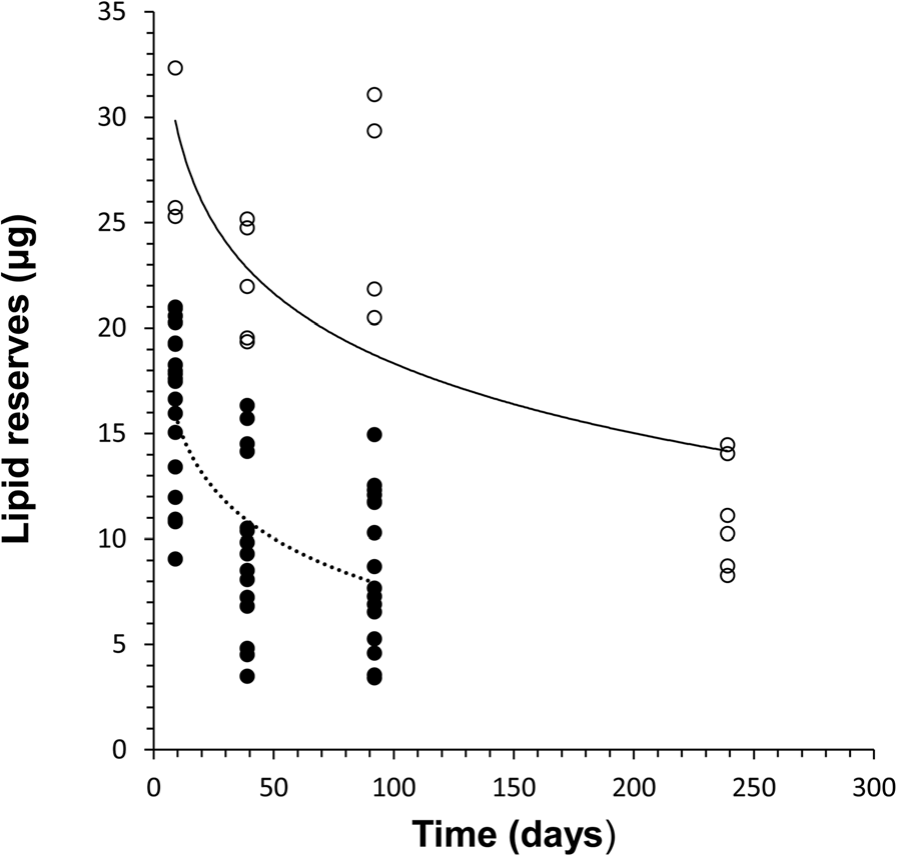
Lipid values (μg), measured by spectrophotometric analysis, in individual nymphal *Ixodes ricinus* collected at a single time point in May 2017 in south-west England, UK, and then starved in a cooled incubator at 15 °C and a relative humifdity of 80% for 9, 39, 92 or 239 days prior to lipid measurement. Two groups are distinguished based on their initial relative lipid values: open symbols and solid line (y = -4.78ln(x) + 40.34, *R*^2^ = 0.55, *P* < 0.05); and closed symbols and dashed line (y = -3.422ln(x) + 23.406, *R*^2^ = 0.44, *P* < 0.05)

For the ticks that were collected in May and maintained in the incubator at 15 °C before analysing after various periods of starvation, overall, lipid values declined significantly over the course of the starvation period (ANOVA: *F*_(3, 71)_ = 3.95, *P* = 0.02). However, cluster analysis showed that the lipid values on day 9 could be divided into two distinct groups with significantly different lipid contents (t-test: *t*_(23)_ = 4.99, *P* < 0.0001), one small group of individuals had relatively high lipid values, the second a more heterogeneous group had significantly lower lipid contents. This discontinuity in the lipid data persisted in the 39 and 92-day samples, but by day 239, the lowest end of the range in lipid values had been lost. The data are therefore plotted as two groups, based on the initial discontinuity in the lipid distribution at day 9. The group with the highest values started with lipid contents of *c.*25–35 μg, which then declined to about 15 μg after 239 days of starvation. In the group with lipid values that started at between 8–25 μg, lipid declined to below an average of 10 μg after 92 days, after which the lowest lipid values seen in early samples were no longer evident; it is therefore assumed that such individuals exhausted their reserves and starved to death (Fig. 3).

### Seasonal pattern of lipid use

**Fig 4:**
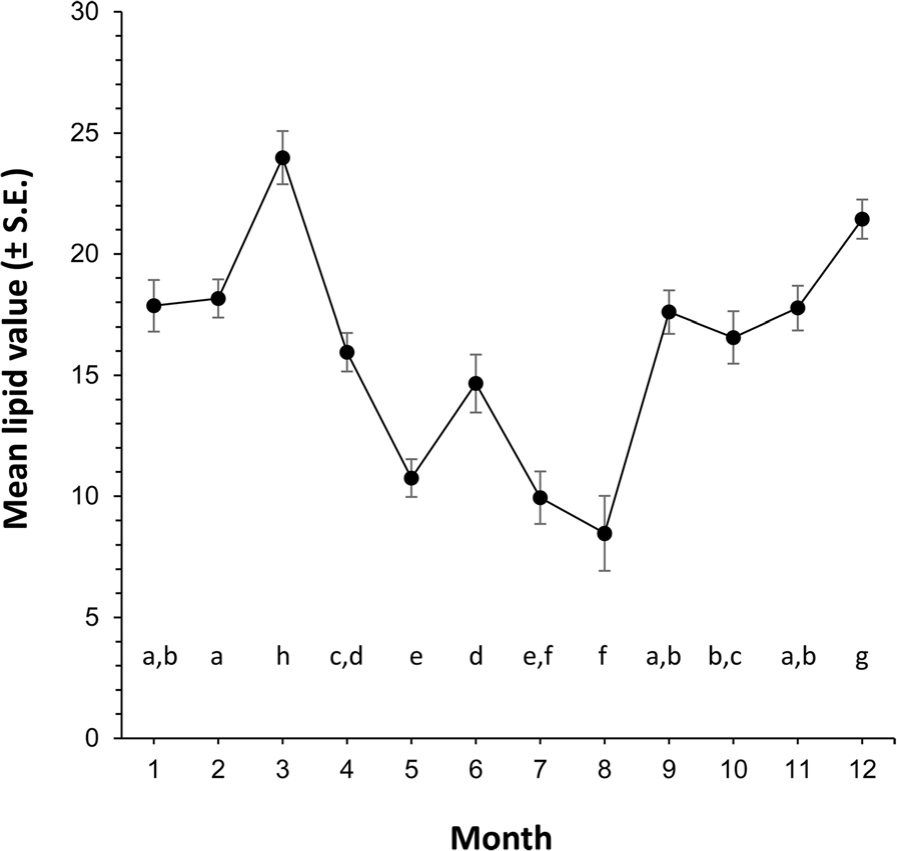
The mean lipid values (± SE confidence intervals) for groups of nymphal *Ixodes ricinus* ticks, measured by spectrophotometric analysis. Ticks were collected by blanket dragging each month in south-west England, UK, during 2015 and 2016. Months 1–12: January-December. Lower case letters above x-axis denote statistically homogeneous groups (*P* < 0.05) as determined by Tukey’s multiple comparisons *post-hoc* tests

**Fig. 5:**
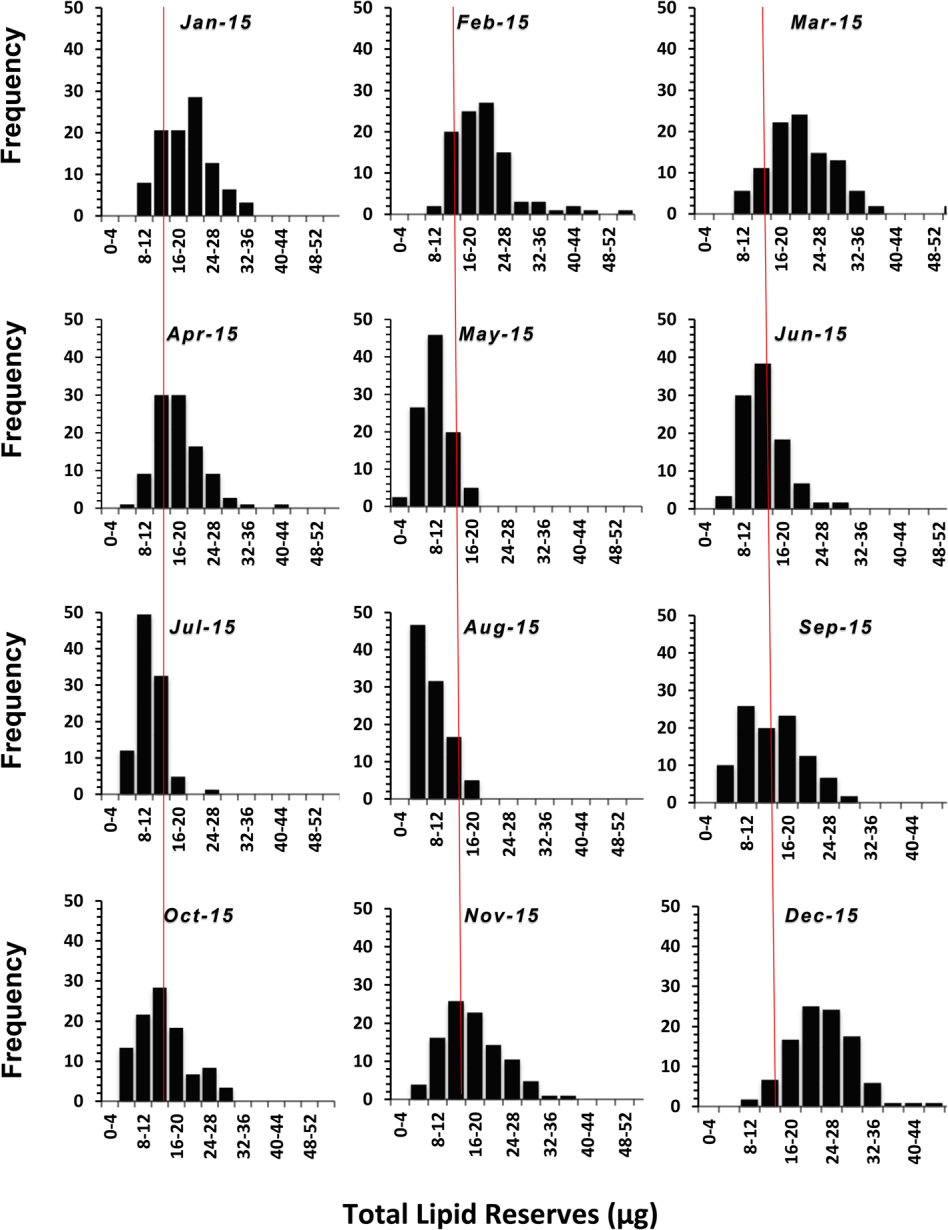
The frequency distribution of observed lipid values (μg), measured by spectrophotometric analysis, in groups of nymphal *Ixodes ricinus* collected by blanket dragging each month in south-west England, UK, during 2015 in 4 μg classes. For reference, the median lipid value for all the samples collected over the two-year period, 14.9 μg, is indicated by the red lines

**Fig. 6:**
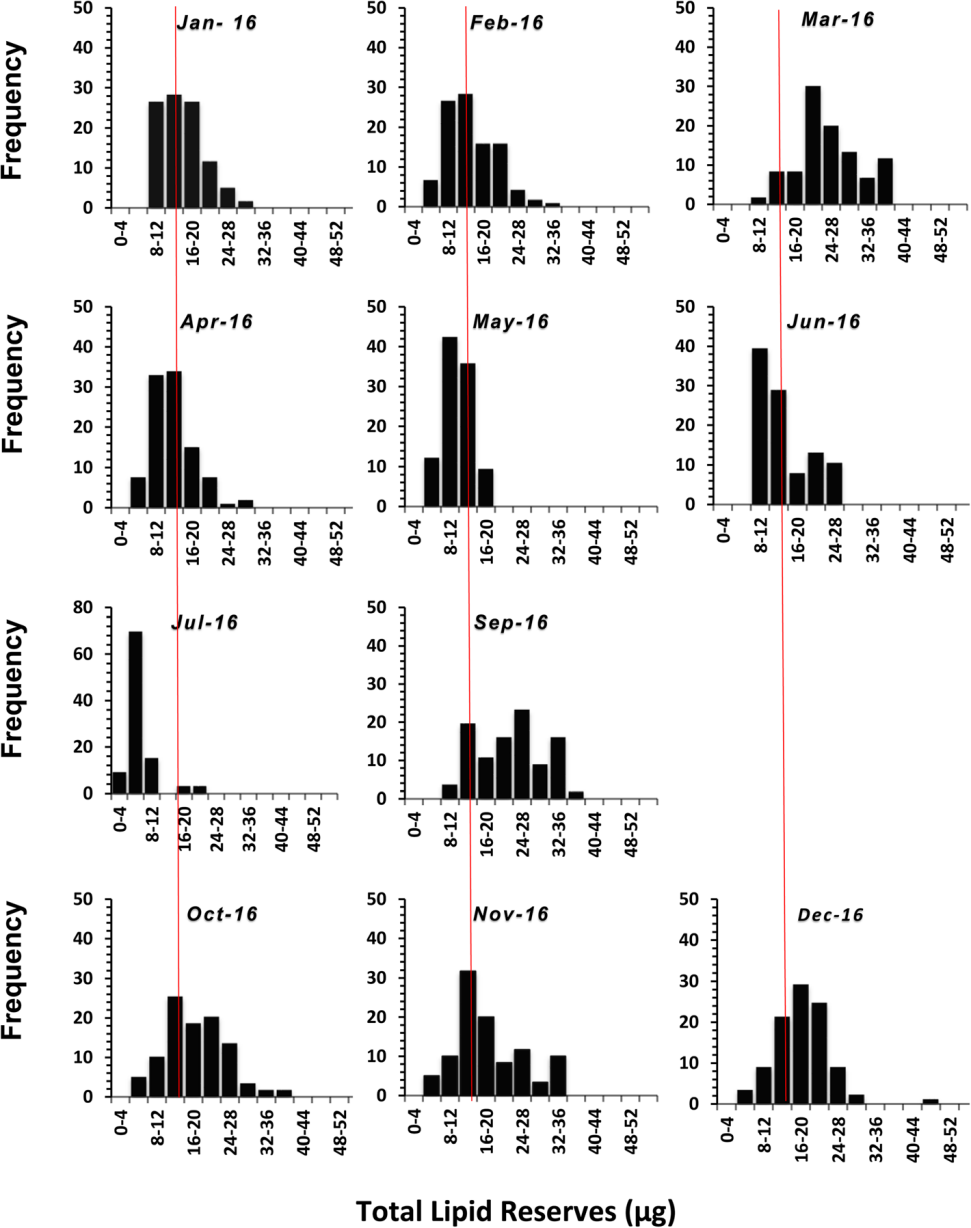
The frequency distribution of observed lipid values (μg), measured by spectrophotometric analysis, in groups of nymphal *Ixodes ricinus* collected by blanket dragging each month in south-west England, UK, during 2016 (excluding August 2016) in 4 μg classes. For reference, the median lipid value for all the samples collected over the two-year period, 14.9 μg, is indicated by the red lines

Lipid values varied significantly over time (ANOVA: *F*_(11, 1843)_ = 74.4, *P* = 0.001; Fig. 4) with values in winter that were significantly higher than values seen in summer. The pattern was consistent over the two years, 2015 (Fig. 5) and 2016 (Fig. 6). Examination of the lipid disributions for individual months shows that starting from January, the nymphs had relatively high lipid reserves, which showed a trend to increase towards March, at which point most of the nymphs had lipid values higher than the median of all the nymphs collected during the two years, which was 14.9 μg (Figs 5, 6). But, from April onwards the nymphs with relatively high lipid values were not present, so that between May and August most of the nymphs had lower lipid reserves. The lipid reserves of the nymphs were again high in September and the trend continued, reaching maximum values in December (Figs. 5, 6).

## Discussion

A spectrophotometric method for lipid measurement, as proposed by Van Handel [13], was evaluated and used to measure lipid reserves in nymphal ticks collected from south-west England, UK. Analysis with known concentration of soybean oil showed that the technique was highly sensitive, capable of accurately detecting lipid to low concentrations. Ticks collected from the field were found to contain reserves of between 5–45 μg of lipid. Comparison with the gravimetric method of lipid estimation that was used by earlier researchers [3, 17, 18], showed that the gravimetric method did not extract all the lipid and that the technique presented here is therefore likely to give a more accurate measure of lipid availability. The method was used to explore the patterns of lipid availability in ticks collected monthly by blanket dragging over a two-year period.

In most of the regions in northern Europe with continental or maritime climates, typified by colder winters and hotter summers, *I. ricinus* shows a bimodal activity pattern, where ticks questing activity peaks in the spring and autumn. Lipid analysis can help to explain the feeding patterns that drive this observed bimodal activity. The lipid reserves in the questing nymphs collected here were lowest in summer for both the years and highest in spring and autumn. Similar seasonal differences in lipid reserves were also reported by Randolph et al. [3]; most nymphs collected between May and August had lower lipid than those collected during the rest of the year. They also found that the nymphs with highest lipid indices appeared in September and October. The data presented here suggest that in spring, the questing population of nymphs is composed of some ticks that fed as larvae the the previous summer and some that fed as larvae in late autumn/winter. The former cohort moulted from the larval to nymphal instar through the spring/summer period and probably started questing in late autumn/winter but were still questing the following spring. Since they fed as larvae several months earlier, in early spring these ticks have relatively low lipid reserves and as shown by the positive skew in the data, these ticks represent the preponderance of the population. The ticks with higher lipid reserves are nymphs that arise from larvae that fed the previous autumn/winter and went through morphogenetic diapause overwinter; they then also moulted to become nymphs and entered the feeding population in the following spring [3]. By midsummer the only nymphs still questing are the ticks with low reserves that have failed to find a blood meal. The nymphs that had low reserves at the start of the year have either fed or starved to death by this stage and the ones that had higher reserves at the start are now close to exhausting their reserves. There is little further recruitment to the feeding population over the summer. By autumn/winter, the increasing numbers of ticks with high lipid reserves again represent those that fed as larvae early in the year, moulted over summer and are now starting to quest as nymphs. Hence, the findings of the present study, indicating that there was a relatively large number of nymphs in spring with high lipid reserves, support the phenology proposed by Gray et al. [19], suggesting that there are separate spring/summer and autumn/winter feeding cohorts.

This explanation is further supported by the laboratory evaluation of the change in lipid percentage with starvation, which showed that for ticks collected in May, a small number had relatively high (*c.*35 μg) and and most relatively low lipid values (*c.*15 μg). The pattern of change in the lipid values suggests that, at 15 °C, the group with relatively low lipid values may have exhausted their reserves and died within about 100 days, since individuals with very low lipid values are lost between days 92 and 240. The data suggest that after about 240 days, the individuals with relatively high lipid values when collected in May, had depleted their reserves to about the starting level of the individuals with relatively low lipid levels in May (*c.*15 μg). Hence, this analysis, allows a quantitative estimate of rates of lipid use, although further work at a range of temperatures would be valuable to allow the temperature-dependent rates of lipid metabolism to be understood in more detail.

## Conclusions

The data illustrate the seasonal change in lipid content of nymphal ticks, reflecting their feeding history and highlight the utility of the spectrophotometric technique for analysis of lipid in ticks in helping to improve our understanding of seasonal activity patterns.
